# Efficacy of Mentalization-based group therapy for adolescents: the results of a pilot randomised controlled trial

**DOI:** 10.1186/s12888-019-2158-8

**Published:** 2019-06-06

**Authors:** Helen Griffiths, Fiona Duffy, Louise Duffy, Sarah Brown, Harriet Hockaday, Emma Eliasson, Jessica Graham, Julie Smith, Alice Thomson, Matthias Schwannauer

**Affiliations:** 1 School of Health in Social Science, The University of Edinburgh, Old Medical School, Teviot Place, Edinburgh, EH8 9AG UK; 2 NHS Lothian Child and Adolescent Mental Health Services, Royal Edinburgh Hospital, Tipperlinn Road, Edinburgh, EH10 5HF UK

**Keywords:** Adolescent, Mentalization, MBT, Group, Self-harm

## Abstract

**Background:**

Mentalization Based Therapy (MBT) has yielded promising outcomes for reducing self-harm, although to date only one study has reported MBT’s effectiveness for adolescents (Rossouw and Fonagy, J Am Acad Child Adolesc Psychiatry 51:1304–1313, 2012) wherein the treatment protocol consisted of an intensive programme of individual and family therapy. We sought to investigate an adaptation of the adult MBT introductory manual in a group format for adolescents.

**Methods:**

The present study is a randomised controlled single blind feasibility trial that aims to (1) adapt the original explicit MBT introductory group manual for an adolescent population (MBT-Ai) and to (2) assess the feasibility of a trial of MBT-Ai through examination of consent rates, attendance, attrition and self-harm. Repeated measures ANOVAs were conducted to examine change over time in independent and dependent variables between groups, and multi level models (MLM) were conducted to examine key predictors in relation to change over time with self-report self-harm and emergency department presentation for harm as the primary outcome variables.

**Results:**

Fifty-three young people consented to participate and were randomised to MBT-Ai + TAU or TAU alone. Five participants withdrew from the trial. Trial procedures seemed appropriate and safe, with acceptable group attendance. Self-reported self-harm and emergency department presentation for self-harm significantly decreased over time in both groups, though there were no between group differences. Social anxiety, emotion regulation, and borderline traits also significantly decreased over time in both groups. Mentalization emerged as a significant predictor of change over time in self reported self harm and hospital presentation for self-harm.

**Conclusions:**

It was feasible to carry out an RCT of MBT-Ai for adolescents already attending NHS CAMHS who have recently self-harmed. Our data gave signals that suggested a relatively brief group-based MBT-Ai intervention may be a promising intervention with potential for service implementation. Future research should consider the appropriate format, dosage and intensity of MBT for the adolescent population.

**Trial registration:**

NCT02771691; Trial Registration Date: 25/04/2016.

## Background

Self-harm is one of the most prominent risk factors for suicide [1, 2]. A recent Scottish study reported that one in six young people had engaged in non-suicidal self-harm whilst one in nine young people had attempted suicide [3]. 10–25% adolescents repeat self-harm behaviours within a year [2, 4]. Adolescents who self-harm are more likely to experience a wide range of psychosocial problems later in life [5]. Self-harm often occurs within the context of a wider, complex mental health presentation, involving the interaction of a variety of social, psychological and cultural factors [4], most commonly with depressive disorders, posttraumatic stress disorder, substance abuse, and borderline personality disorder (BPD) [2, 6, 7]. A majority (62.4%) of those who die by suicide aged 35 years and younger were found to have presented to health services the year preceding their death [8]. This provides significant potential for early intervention to decrease self-harm and suicide prevalence, and also to prevent the escalation of parallel emotional distress and functional impairments.

Difficulties such as emotion dysregulation and decreased social functioning are predictive of self-harm in young people [9, 10]. It has also been suggested that self-harm can be understood in the context of reduced and or a temporary loss of mentalizing capacity, particularly in the context of interpersonal stress. Mentalizing is the mental activity that enables us to perceive and interpret human behaviour in terms of intentional, motivational and emotional mental states (e.g., needs, desires, feelings, beliefs, goals, purposes, and reasons) [11, 12]. Self-harm is in part presumed to be the result of a failure to make sense of social experience, resulting in reduced adaptive coping and impulsive behaviours including self-harm [12, 13]. Indeed, mentalization was recently found to be the only independent variable to predict higher risk of suicide in psychiatric adult inpatients [14].

A number of approaches with little to no evidence are currently considered to be best-practice within clinical settings [15]. The need to further develop and replicate trials for self-harm in adolescence has been repeatedly highlighted throughout the literature [16–19]. Two recent systematic reviews evidenced promising effects for three therapeutic interventions namely cognitive behavioural therapy, dialectal behaviour therapy (DBT), and MBT [17, 19]. MBT, which was initially developed for the treatment of adults with a diagnosis of borderline personality disorder (BPD), directly addresses the fragile mentalizing capacity that is a core feature of BPD [20] and other complex pathology. Within an adult BPD population, MBT reduced self-harm, emotional distress and inpatient stays, and improved social function in comparison to treatment as usual [21] with continued improvements at 18 months [22]. MBT was also associated with reduced self-harm in an adult outpatient setting [23].

In relation to the adolescent population, MBT-A was more effective than treatment as usual for young people who self-harm [13]. The treatment protocol involved weekly individual MBT-A sessions and monthly MBT family therapy (MBT-F) delivered over 12 months. In a non-controlled pilot, self-reported self-harm reduced in the context of an intensive MBT group programme for adolescents with BPD or subthreshold BPD that included introductory psychoeducational group sessions, 34 sessions of MBT group-therapy and 7 sessions of MBT-parents (MBT-P) [24]. Psychoeducational group sessions aim to increase understanding of mentalizing, emotion regulation and so on, whilst group therapy sessions are more process-focussed, aiming to facilitate mentalizing between group participants. It is not known which modalities of MBT (individual, group, family, parents) are most effective or instrumental in any observed clinical change. Furthermore, little is known about the required intensity of intervention.

In our own version, we adapted the introductory MBT group with adults (MBTi) [25] for use with an adolescent population (MBT-Ai). A key aim is the development of MBT knowledge about underlying principles and concepts of mentalization. Whilst MBTi for previous trials was designed as a psychoeducation precursor to a combination of MBT individual and group therapy, our MBT-Ai programme incorporates a number of experiential tasks and role plays to encourage the application of mentalization principles to common interpersonal dilemmas.

The present study aims to evaluate the feasibility and effects of 12 week MBT-Ai for the reduction of self-harm and crisis presentations in a group of young people already receiving treatment within specialist NHS Child and Adolescent Mental Health Services (CAMHS).

## Methods/design

We report on the proportion of potential participants who consented to participate in the trial, group attendance, ability to follow-up participants, trial withdrawals, and serious adverse events. We also report on the clinical characteristics of the trial participants at entry, end of treatment and follow-up, including any group differences. Study methodology will be reported in accordance with the Standard Protocol Items: Recommendations for Interventional Trials (SPIRIT) statement and guidelines [26]. Further information about the development of the group protocol and trial methodology is available elsewhere [27].

### Study design

This study is a two-arm, single (rater) blind, randomised controlled trial registered with ClinicalTrials.gov (Trial registration: NCT02771691).

### Setting

The recruitment area serves a population of approximately 160,000 young people under 18 years and provides a range of outpatient and more specialist services. Staff from Tier 4 services, which include day programmes and assertive outreach teams, have received training in Adaptive Mentalization-Based Integrative Treatment (AMBIT) [28]. Four trained MBT therapists from this service developed and piloted the MBT-Ai group manual under supervision provided by the Anna Freud Centre.

### Participants

Inclusion criteria were as follows: (1) Aged 12–18 years (2) self-harm behaviour in the past 6 months (3) in receipt of CAMHS treatment (4) competent and willing to provide written, informed consent. Exclusion criteria were: (1) severe learning disability or pervasive developmental disorder (2) acute psychotic episode (3) eating disorder in the absence of self-harm (4) non-English speaking (5) current involvement in other ongoing treatment research.

### Procedure

Research assistants blind to randomisation conducted assessments of primary and secondary outcomes and liaised regularly with the relevant clinical teams. Masking was maintained using a wide range of measures. Trial unblindings were reported to the Trial Manager who implemented corrective action if necessary. Key clinicians identified potential participants, offered them a participant information sheet and invited them to take part. Self-referrals were also accepted. The treating clinicians confirmed they met criteria and that all young people had capacity to consent.

We applied the principle of direct consent for all potential participants. During the recruitment/consenting process the researcher ensured that the young person was fully informed of the randomisation process and their chances of receiving MBT-Ai group therapy.

Once written consent had been obtained, baseline measures were completed. Primary and secondary outcomes were carried out within a single 30-min session at each time point wherever possible. Case note review was completed at the end of treatment.

Participants were withdrawn from the trial if they withdrew consent. A distinction was made as to whether the individual was withdrawing consent from further trial treatment only or withdrawing from trial treatment and follow-up.

### Randomisation/treatment allocation

Randomisation (at the individual level) was independent and concealed, using randomised-permuted blocks adjusted to permit access to group treatment without undue delay. Group allocation remained concealed until completion of self-report ratings.

### Study arms

#### Mentalization based treatment for adolescents (MBT-Ai) group therapy

Key aims of MBT-Ai were to encourage emotional literacy; introduce concepts of mentalization, attachment and emotion regulation; facilitate reflection on interpersonal relationship patterns; and explore how these concepts affect emotional expression, behaviour and mental health. Up to 12 sessions of MBT-Ai were delivered by trained MBT therapists, who were highly experienced clinical psychologists under the supervision of an MBT accredited supervisor, to up to 10 young people per group. Our groups were 1.25 h long, and always started with a warm-up exercise to encourage group participation. We used worksheets, DVD clips and specific case material providing real life age-appropriate examples as a way to enhance learning and facilitate group discussion. The application of mentalization techniques to common daily dilemmas was encouraged throughout. Technical language was simplified where possible e.g. “avoidant attachment” was replaced with “distant style of relating”. The manual is available from the corresponding author on request.

Fidelity to protocol and adherence to the principles of MBT was checked by means of audiotape ratings which were rated by an MBT accredited supervisor using an adherence tool adapted for our MBT-Ai group format and fed back into supervision.

#### Treatment as Usual (TAU)

TAU was delivered according to national and local service protocols and guidelines. Given that we recruited from a population already attending either tier 3 or tier 4 CAMHS, the young people may be experiencing a number of difficulties in addition to self-harm behaviour. Depending on the presenting problems, treatment could therefore consist of any combination of key worker input, psychological/psychosocial intervention and medication. Local services consist of multidisciplinary teams that may include child and adolescent psychiatrists, social workers, clinical psychologists, community psychiatric nurses, occupational therapists and community mental health workers. Tier 3 services are offered in community mental health settings on an outpatient basis. Tier 4 provides tertiary level services including intensive community treatment, day programmes and an inpatient unit. In order to establish the parameters of the TAU package [29], service use was measured using an adapted version of the Client Service Receipt Inventory (CSRI) [30] which provided a summary of health services accessed by the young people over the study period. Referrers were not asked to withhold any treatment as this was considered unethical.

### Data collection

We collected data about the proportion of young people who consented to randomisation, study withdrawal and the occurrence of any serious adverse events. An event was considered to be a serious adverse event if it resulted in one of the following outcomes: a) death b) was life-threatening c) inpatient hospitalisation or prolongation of existing hospitalisation d) persistent or significant disability or incapacity. For the MBT-Ai arm only, group attendance and attrition rates were also monitored.

#### Outcome measures

Our primary outcome was self-harm post-treatment as assessed by:

1. The self-harm subscale of the Risk-Taking and Self-Harm Inventory for Adolescents (RTSHI) [31]: a self-report measure designed to assess adolescent risk-taking and self-harm in community and clinical settings. In the original study, both self-harm and risk-taking factors of the RTSHI demonstrated high internal consistency, test-retest reliability and sufficient validity in youth.
2. Self-harm related hospital use as reported by emergency department presentation in NHS electronic records.

Participants also completed the following self-report measures:

- Risk-taking as measured by the risk-taking subscale of the Risk-Taking and Self-Harm Inventory (RTSHI) [31].
- Emotional distress (anxiety and depression) as measured by the Revised Child Anxiety and Depression Scale (RCADS) [32]. The RCADS has evidenced reliability and validity in both clinical [33] and school-based samples of children and adolescents [34, 35].
- Mentalization as measured by the self-report Reflective Functioning Questionnaire for Youths (RFQ-Y). This measure has demonstrated adequate internal consistency, convergent validity and reliability in an adolescent sample [36].
- Emotion regulation as measured by the Difficulties in Emotion Regulation Scale (DERS) [37]. This measure has evidenced high internal consistency, adequate validity and good test-retest reliability in young adults [37] and adolescents [38].
- Interpersonal sensitivity as measured by the Interpersonal Sensitivity Measure (ISM) [39]. Validity and reliability has been evidenced in populations diagnosed with depression and anxiety [39, 40].
- Borderline Traits as measured by the 11-item short version of the Borderline Personality Features Scale for Children (BPFSC) [41]. In the original study, satisfactory construct and criterion validity in adolescent samples were established.
- Attachment as measured by the 12-item short version of the Experiences in Close Relationships Scale–Revised Child version (ECRS-RC), shown to have excellent validity and reliability in youth samples [42].

All measures were administered blind by the research assistants at baseline, 12, 24 and 36 weeks. In addition, the RFQ-Y was administered at 6, 18 and 30 weeks. For self-harm related emergency department use, the research assistants reviewed NHS electronic records after the last face to face assessment had been conducted in order to maintain blinding. Details of all crisis service use (e.g. presentation at emergency department or other mental health emergency service provision and related hospital in-patient admissions) were recorded on a pre-specified proforma along with a description of the self-harm and confirmation that the hospital admission was in relation to that self-harm.

### Power and statistical analysis

In this feasibility trial the sample size was not predetermined. We aimed for sufficient data to report on response rates, follow-up rates, safety information and attrition, as well as the clinical characteristics of our study population at the beginning and end of the trial and follow-up.

The majority of our reporting is descriptive. However, in order to assess whether the receipt of MBT-Ai plus TAU compared to TAU alone, leads to reduced self-harm and crisis presentations we also carried out intention-to-treat analyses to determine indicative treatment effects on the primary and secondary outcome measures, adjusting for pre-specified baseline covariates. A correlation matrix was conducted to explore the outcome measures to see if there were strong treatment signals within these sub scales. Subsequently, three multi level models (MLM) were constructed, adjusting for baseline measures to assess the treatment effects on the primary and secondary outcome measures and the key predictors of the trajectories of these treatment effects over time.

## Results

### Demographic and clinical data

**Fig. 1:**
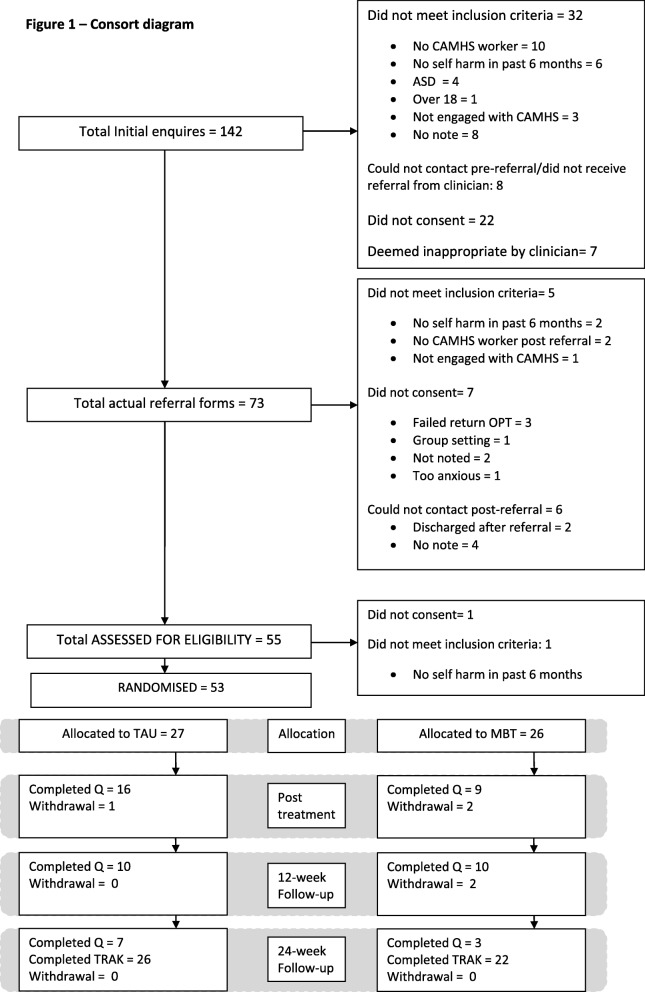
Consort diagram

There were 73 referrals to the trial. Of these, 53 (73%) young people consented to participate and were randomised to either MBT-Ai plus TAU or TAU alone. Reasons for non-randomisation are provided in Fig. 1.

At entry to the study, there were no significant between group differences on demographic or service usage variables (see Table 1). Our sample was slightly older than the original MBT trial for adolescents [13] with a mean age of 15.5 years compared to 14.7 years. Our population was 77% female compared to 85% in the original trial. The MBT-Ai plus TAU group reported significantly higher scores on the BPD total score (t(46) = − 2.016, *p* = .050) as assessed on the BPFSC. Using the recommended cut-off for this scale [41] 55% of participants in the treatment arm warranted further assessment for borderline personality disorder compared to only 15% of the control arm. There were no other significant differences on baseline measures between the two groups. The mean score of self-reported self-harm at baseline was 27.6 (SD = 11.9) compared to 15.78 (SD = 9.84) reported for a clinical sample recruited to an RCT for self-harm reported in the original validation paper [31]. The higher levels in our sample may reflect rises in help-seeking for self-harm behaviour reported, particularly for young women, over the last decade (e.g. [3]). Not surprisingly since we recruited a group who were already receiving treatment from CAMHS, the mean total score for both groups on the RCADS placed our total population firmly within the clinical range [33]. Given that our inclusion criteria were behaviourally rather than diagnostically informed, we did not collect information about medication at baseline. However, whilst contact with a psychiatrist does not equate to being prescribed medication, our service usage data highlighted that 16 young people (33%) had had one or more appointments with psychiatry, eight young people from each arm.

**Table 1:** Demographic, Service Use and Clinical Characteristics at Baseline

Characteristics at Baseline	TAU	MBT-A
Female, n/N (%)	21/26 (80.8)	17/22 (77.3)
Age, years, mean (SD)	15.7 (1.4)	15.4 (1.3)
Living with parent/guardian or other family, n/N (%)	22/26 (84.6)	18/22 (81.8)
Enrolled in formal education^a^, n/N (%)	22/26 (84.6)	17/22 (77.3)
Ethnicity white Scottish, n/N (%)	18/26 (69.2)	15/22 (68.2)
Socioeconomic status = <60th percentile, n/N (%)	11/26 (42.3)	10/22 (45.5)
Number of appointments 6-months pre-treatment, mode (range)	14.5 (1–80)	11.0 (2–81)
Number of appointments between treatment and 24-week follow-up, mode (range)	9.0 (0–104)	17.0 (0–108)
Years since initial referral to CAMHS, n/N (%)		
< 1 year	7/26 (26.9)	9/22 (40.9)
1–2 years	13/26 (50.0)	3/22 (13.6)
2–3 years	3/26 (11.5)	5/22 (22.7)
3 years +	2/26 (7.7)	5/22 (22.7)
Unknown	1/26 (3.8)	0/22 (0)
Self-reported self-harm at baseline	26.8 (11.8)	28.3 (11.9)
Self-reported risk taking at baseline	12.7 (7.0)	12.6 (9.3)
Self-harm related A + E presentation 6 months pre-baseline, mean (range)	1.08 (0–7)	0.73 (0–4)
RCADS	79.8 (24.9)	91.1 (22.0)
RFQ	8.1 (0.8)	8.3 (0.8)
DERS	133.8 (21.9)	140.4 (17.9)
ERC	6.9 (1.9)	7.7 (2.2)
BPD	28.4 (5.6)*	32.0 (6.9)*
ISM^b^	82.5 (12.3)	85.9 (7.4)

^a^Includes high school and/or college; ^b^only 18 TAU and 13 MBT completed baseline assessment *significant at *p* ≤.05

Five participants withdrew from the trial. Post-treatment we collected self-report data from 50% participants who remained in the trial and 42% of those at 12 week follow-up. Patient records data reporting self-harm related hospital use taken at 36 weeks was available for all trial completers. There were 5 adverse events involving 4 young people, but none of these were considered to be trial related.

Twenty-six young people were randomised to the MBT-Ai plus TAU arm of the trial and were offered the group intervention. The average number of sessions attended was 5.3 (ranging from 0 to 12). Six (27.3%) young people did not attend any sessions; five (22.7%) attended 1–6 sessions; and 11 (50%) young people attended 50% or more.

### Changes in self-harm & clinical variables

Intent to treat (ITT) analytic strategy was employed in order to reduce bias in treatment effects resulting from missing data. Missing values at an item-level of < 15% were imputed based on a multiple imputation model. Last observation carried forward was the conservative imputation strategy used for questionnaire level missing data.

Normality of distributions was tested using the Shapiro-Wilk test. There was indication that three of the RTSHI scores (RT at post, 12- and 24-week follow-up) were skewed, but none of the SH measures. In order to examine trajectories over time we conducted multi-level modelling (MLM) to examine change over time (from baseline to post-treatment and follow-up) with self-reported self-harm and ED admissions due to self-harm as dependent variables and baseline self-reported risk-taking, emotion regulation and mentalization entered as independent fixed effect predictors.

There was a significant decrease in both self-reported self-harm and emergency department presentation but not risk-taking behaviour from baseline to post-treatment, with an observed power of 0.767 and 0.845 respectively. See Table 2 for results. However, there was no difference in change over time between groups. In order to detect a small effect over time and between group in our sample power of 0.875 would be needed for self reported self harm and 0.998 for emergency presentations.

**Table 2:** Emergency department presentation and self-reported self-harm, Change over time

	Pre-treatment		Post-treatment		12-wk follow-up		24-wk follow-up		Spher	Change over time		Time X Group	
Continuous measure	TAU (*n* = 26)	MBT-A (*n* = 22)	TAU (*n* = 26)	MBT-A (*n* = 22)	TAU (*n* = 26)	MBT-A (*n* = 22)	TAU (*n* = 26)	MBT-A (*n* = 22)	W	MS	F	MS	F
Self-Harm subscale (RTSHI) Mean (SD)	26.82 (11.80)	28.32 (11.89)	23.12 (12.28)	26.00 (12.57)	22.93 (12.35)	24.41 (12.52)	22.74 (13.04)	24.50 (13.88)	.518***	225.02	6.29**	7.17	.201, NS
Risk Taking subscale (RTSHI) Mean (SD)	12.66 (7.02)	12.61 (9.28)	12.88 (8.28)	12.78 (9.64)	13.21 (8.52)	12.82 (9.94)	13.29 (8.27)	12.65 (10.25)	.601***	1.406	.210, NS	.888	.133, NS
Total (RTSHI) Mean (SD)	39.48 (16.94)	40.93 (18.08)	36.00 (18.80)	38.78 (19.65)	36.14 (19.67)	37.24 (20.22)	36.03 (19.91)	37.16 (21.90)	.477***	183.34	2.83, NS	10.01	.154, NS
Self-Harm ED Presentation Mean (range)	1.08 (0–7)	0.73 (0–4)	0.23 (0–2)	0.36 (0–2)	0.54 (0–3)	0.23 (0–2)	0.35 (0–4)	0.09 (0–1)	.527***	6.49	9.55***	0.84	1.24, NS

*Abbreviations: ED* Emergency Department, *RTSHI* Risk-Taking and Self-Harm Inventory

*p < .05,**p < .01,***p < .001

Change over time was also observed on several of the self-report questionnaire variables, namely social anxiety, emotion regulation and borderline personality features, as illustrated in Table 3. Similarly, there were no significant group differences over time.

**Table 3:** Self-report variables, change over time

	Pre-treatment		Post-treatment		12-wk follow-up		24-wk follow-up		Spher.	Change over time		Time X Group	
Continuous measure	TAU (*n* = 26)	MBT-A (*n* = 22)	TAU (*n* = 26)	MBT-A (*n* = 22)	TAU (*n* = 26)	MBT-A (*n* = 22)	TAU (*n* = 26)	MBT-A (*n* = 22)	W	MS	F	MS	F
RCADS (SP)	18.68 (6.29)	19.65 (5.8)	17.72 (6.35)	19.49 (5.81)	18 (5.97)	19.38 (6.25)	18.81 (6.01)	18.96 (6.72)	.35***	4.36	.856, NS	8.50	1.66, NS
RCADS (PD)	14.65 (6.37)	16.69 (6.13)	12.8 (5.99)	15.73 (6.36)	13.85 (6.18)	14.23 (6.31)	13.46 (6.47)	14.92 (5.93)	.19***	48.25	2.755, NS	23.91	1.37, NS
RCADS (MD)	19.12 (6.35)	21.12 (4.43)	18.15 (6.57)	20.39 (4.74)	17.81 (6.65)	19.89 (5.64)	18.49 (6.96)	20.07 (5.72)	.37***	21.83	2.41, NS	1.55	.171, NS
RCADS (SA)	8.49 (4.36)	10.3 (5.5)	6.98 (4.03)	9.96 (5.64)	7.21 (4)	9.51 (5.47)	7.25 (3.73)	9.69 (5.54)	.31***	18.92	4.17*	4.73	1.04, NS
RCADS (GA)	10.52 (3.95)	12.73 (3.57)	9.77 (3.72)	12.64 (4.09)	10.27 (3.73)	12.18 (4.2)	10.38 (3.82)	12.55 (3.96)	.22***	3.67	.523, NS	3.76	.534, NS
RCADS (OC)	8.33 (3.08)	10.59 (3.4)	7.73 (3.34)	9.78 (3.48)	8.29 (3.26)	9.2 (4.07)	8.38 (3.53)	9.37 (3.87)	.34***	8.21	1.62, NS	8.88	1.75, NS
RCADS Anx	71.46 (22.74)	80.48 (19.99)	65.42 (22.4)	78.21 (21.48)	67.14 (22.05)	76.56 (25.24)	68.4 (21.61)	77.55 (24.91)	.151***	347.28	2.15, NS	73.78	.457, NS
RCADS Inter	79.79 (24.88)	91.07 (22.04)	73.15 (24.24)	87.99 (23.67)	75.43 (24.18)	85.76 (28.05)	76.78 (23.84)	86.93 (27.58)	.151***	480.53	2.27, NS	109.16	.515, NS
RFQY ScaleA	4.18 (0.49)	4.19 (0.51)	4.18 (0.49)	4.29 (0.72)	4.21 (0.53)	4.46 (0.79)	4.14 (0.61)	4.36 (0.71)	.404***	.287	1.40, NS	.210	1.03, NS
RFQY ScaleB	3.96 (0.45)	4.12 (0.38)	3.96 (0.52)	4.05 (0.32)	3.95 (0.55)	4.1 (0.38)	3.92 (0.56)	4.08 (0.33)	.227***	.037	.353, NS	.022	.212, NS
RFQY Total	8.14 (0.79)	8.31 (0.76)	8.14 (0.78)	8.34 (0.82)	8.16 (0.76)	8.56 (0.88)	8.06 (0.79)	8.44 (0.77)	.389***	.292	.81, NS	.266	.736, NS
DERS NA	21.42 (5.06)	23.59 (5.24)	20.23 (6.11)	21.32 (5.5)	20.62 (6.54)	21 (5.49)	21.38 (7.94)	21.95 (5.56)	.667**	40.38	3.27*	9.55	.773, NS
DERS Goal	21.58 (2.84)	21.82 (2.79)	20.85 (3.29)	20.95 (3.2)	20.5 (2.96)	20.95 (3.24)	20.42 (3.49)	21.45 (3.08)	.489***	13.09	2.22, NS	2.93	.495, NS
DERS Impulse	22.38 (4.84)	23.59 (4.85)	21.88 (5.3)	22.82 (5.21)	21.33 (5.54)	22.33 (5.54)	21.27 (5.1)	23.5 (5.27)	.800	10.67	1.72, NS	4.36	.704, NS
DERS Aware	20.92 (4.51)	21.5 (4.82)	21.76 (4.62)	21.5 (4.03)	21.31 (4.68)	20.77 (4.45)	20.92 (4.77)	21.41 (4.17)	.522***	4.46	.612, NS	5.14	.705, NS
DERS Strat	30.33 (6.89)	31.86 (6.06)	29.61 (7.24)	30.18 (6.17)	29.12 (7.47)	30.04 (6.94)	29.27 (7.86)	30.82 (6.8)	.674**	26.00	2.89*	3.46	.385, NS
DERS Clarity	17.21 (4.71)	18.05 (3.37)	16.48 (4.7)	17.91 (4.29)	17.03 (4.32)	17.59 (4)	17.95 (5.46)	18.14 (4.02)	.279***	12.26	1.26, NS	5.82	.60, NS
DERS Total	133.85 (21.9)	140.41 (17.9)	130.81 (23.29)	134.68 (20.18)	130.05 (23.83)	132.7 (21.45)	131.37 (23.67)	137.28 (19.88)	.685***	365.05	3.22*	48.80	.431, NS
ECRS Anxiety	3.04 (1.53)	3.74 (1.78)	3.09 (1.79)	3.41 (1.81)	2.82 (1.87)	3.33 (1.76)	2.81 (1.83)	3.5 (1.92)	.367***	1.36	2.07, NS	.598	.913, NS
ECRS Avoid	3.85 (0.65)	3.92 (0.88)	3.84 (0.46)	3.83 (0.79)	3.85 (0.5)	3.8 (1.04)	3.81 (0.56)	3.9 (1.05)	.299***	.050	.153, NS	.098	.301, NS
ECRS Total	6.89 (1.85)	7.66 (2.24)	6.94 (1.99)	7.25 (2.12)	6.67 (2.13)	7.13 (2.31)	6.62 (2.09)	7.4 (2.52)	.334***	2.044	1.52, NS	1.152	.859, NS
BPF total	28.42 (5.59)	32.05 (6.86)	27.5 (5.88)	29.59 (6.91)	27.42 (6.26)	28.77 (6.76)	27.42 (6.33)	29.09 (6.55)	.418***	68.37	5.35**	17.87	1.40, NS

*Abbreviations: RCADS* Revised Child Anxiety and Depression Scale (*SP* social phobia, *PD* panic disorder, *MD* depression, *GA* generalized anxiety, *SA* separation anxiety, *OC* obsessive-compulsive disorder, *Anx* anxiety total, *Inter* internalizing total), *RFQY* Reflective Functioning Questionnaire for Youths, *DERS* Difficulties in Emotion Regulation Scale (*NA* nonacceptance, *Goal* difficulties engaging in goal directed behavior, *Impulse* impulse control difficulties, *Aware* lack of emotional awareness, *Strat* limited access to emotion regulation strategies, *Clarity* lack of emotional clarity), *ECRS* Experiences in Close Relationships Scale–Revised Child version, *BPF* Borderline Personality Features Scale for Children

*p < .05,**p < .01,***p < .001

### Interaction between clinical variables

In order to explore any further signals about interactions between our variables, non parametric bivariate correlations were examined between questionnaire outcomes and both self-reported and hospital presentation self-harm outcomes at baseline and at follow-up. Reported self-harm at follow-up was associated with baseline reported self-harm, risk-taking and poor emotion regulation. Emergency department presentations at follow-up were associated with baseline reported self-harm, emergency department presentations and reflective function at baseline (Table 4).

**Table 4:** Correlation Matrix: All self-harm

	Self-harm BL	Self-harm FU	RCADS BL	RFQY BL	DERS BL	ECR Anxiety BL	ECR Avoidant BL	BPF BL	Risk-taking BL	Hosp. Self-harm BL	Hosp. Self-harm FU
Self-reported Self-harm BL	1	.814**	.267	−.150	.392**	.194	.185	.048	.513**	.337*	.308*
Self-reported Self-harm FU		1	.117	−.212	.332*	.108	.082	.129	.460**	.229	.282
RCADS BL			1	.365*	.522**	.522*	.506**	.569**	.055	−.074	.007
RFQY BL				1	−.046	.163	.192	.341*	−.150	−.433**	−.400**
DERS BL					1	.388**	.285*	.375**	.194	.068	.003
ECR Anxiety BL						1	.335*	.280	.035	.003	.052
ECR Avoidant BL							1	.142	−.012	.128	.156
BPF BL								1	.133	−.327*	−.248
Risk-taking BL									1	.000	.029
Hosp. Self-harm BL										1	.847*
Hosp. Self-harm FU											1

*Abbreviations: BL* Baseline, *FU* follow-up, *RCADS* Revised Child Anxiety and Depression Scale, *RFQY* Reflective Functioning Questionnaire for Youths, *DERS* Difficulties in Emotion Regulation Scale, *ECRS* Experiences in Close Relationships Scale–Revised Child version, *BPF* Borderline Personality Features Scale for Children, Hosp. Self-*Harm* hospital related self-harm

*p < .05,**p < .01,***p < .001

### Trajectories of treatment effects: self-harm

We utilized multi-level modelling (MLM) conducted with R (R Development Core Team, 2017) to examine change over time (from baseline to post-treatment and follow-up) with self-reported self-harm and ED admissions due to self-harm as dependent variables and baseline self-reported risk-taking, emotion regulation and mentalization entered as independent fixed effect predictors. There are several advantages to utilizing MLM that make the data analytic method appropriate for the current investigation. One of these benefits includes the ability for data to be modelled at two different levels: Level 1 describes within-individual change over time (e.g., trajectory of self harm and ED admissions), and Level 2 allows the prediction of between individual-level differences in this change (e.g., group). An additional advantage to using MLM is that the approach accounts for missing data at Level 1 by estimating the trajectory using all existing data for that participant. This benefit is crucial in any longitudinal data analyses so as not to exclude participants with partial data on the dependent variables. In addition to the approach’s benefits, MLM allows to control for baseline scores of each measure when investigating change in each construct over time.

Prior to conducting the proposed MLM models, the appropriateness of using this analytic approach was examined by building two null models. Each null model separately tested each of our dependent variables (i.e., self reported self harm and ED admissions due to self-harm) to determine the potential for correlated error and the need for linear mixed modelling. In both cases, the intraclass correlation coefficient (ICC) was significant, indicating that a multilevel model was appropriate and necessary. For self reported self harm, the correlation in the construct across time within participants was 0.31 and for ED admissions, it was 0.58.

We specified Level-1 intercepts and slopes as random given expected within-person variability in baseline scores and change in constructs over time. We tested a total of three models. The trajectory models were first estimated to evaluate systematic linear changes over time.

For every model that was tested, the intercepts for self reported self harm and Hospital admissions due to self-harm were significant, indicating that baseline scores for each of these variables was different across individuals (see Table 5).

**Table 5:** Results of MLM of self reported self harm and self harm related hospital admissions

	Model1	Model 2	Model 3
Self harm			
Intercept	26.83 (1.77)***	14.60 (3.63)***	34.53 (10.88)**
Time	−0.66 (0.15)***	−0.63 (0.12)***	−0.65 (0.12)***
Baseline self harm		0.51 (0.08)***	1.53 (0.33)***
Risk taking		0.77 (0.08)***	0.60 (0.08)***
Emotion regulation		0.14 (0.02)***	0.12 (0.02)***
Mentalization			2.83 (1.25)*
mentalization x BL SH			−0.16 (0.03)**
Log likelihood	− 928.32	−852.20	− 848.09
AIC	1864.65	1718.40	1714.19
ED admissions			
Intercept	0.46 (0.16)*	0.48 (0.31)	1.31 (0.82)
Time	−0.07 (0.02)*	− 0.07 (0.02)**	− 0.07 (0.02)*
Baseline self harm		0.04 (0.02)**	0.04 (0.01)**
Risk taking		−0.02 (0.01)*	−0.03 (0.01)*
Mentalization			−0.21 (0.09)*
Log Likelihood	− 397.122	−397.15	− 395.91
AIC	802.24	806.66	805.82

Values in parentheses represent standard errors. Significance: ***p < .001; **p < .01; * *p* < .05

Model 1 for for self reported self harm and Hospital admissions due to self-harm shows significant change over time. Model 2 adds significant baseline predictors of baseline self harm, risk taking and emotion regulation to the model demonstrating their significant contribution for both self reported self harm and Hospital admissions due to self-harm.

In the final model 3, while controlling for the effects of self harm at baseline, self reported self harm followed a linear trajectory with scores decreasing over the course of time (β = − 0.66, SE = 0.15, *p* < .001). Risk taking was significantly related to the construct’s linear trajectory (β = 0.77, SE = 0.08, *p* < .001); emotion regulation (β = 0.14, SE = 0.02, *p* < .001) and mentalization (β = 2.83, SE = 1.25, *p* < .001) was associated significantly with self reported self harm. Furthermore, there was a significant interaction between baseline or pre treatment self harm and mentalization (β = − 0.16, SE = 0.04, *p* < .001).

Hospital admissions due to self harm followed a quadratic trajectory with scores decreasing over time (β = − 0.07, SE = 0.02, *p* = .009). Risk taking was significantly related to the construct’s linear trajectory (β = − 0.03, SE = 0.01, *p* = .01) and mentalization (β = − 0.21, SE = 0.08, *p* = .01) was associated significantly with reduced ED admissions self harm.

## Discussion

MBT-A has shown promising effects [13]. However it is not known which MBT modality (individual, group, family), or combination of modalities, is most effective, nor how long or intensive therapy needs to be order to confer benefit.

We found that a pilot RCT of group-based MBT-Ai to reduce self-harm is both feasible and acceptable to participants. Clinicians expressed significant interest in referring to the trial, perhaps reflecting a lack of viable treatment options for these young people. Our broad inclusion criteria may also have contributed to our successful recruitment strategy, facilitating easy referral from clinicians. Not surprisingly for a clinical sample, our population experienced high levels of distress and self-harm behaviour. Over half of the sample warranted further assessment of borderline personality features on the basis of their scores on a self-report measure. Nonetheless, having recruited on the basis of self-harm behaviour from all teams within CAMHS, our population was somewhat heterogeneous. Ensuring that young people understood that randomisation may result in no additional treatment seemed to mitigate against any undue attrition from the control group. During a previous pilot phase young people had expressed a strong preference for groups to have closed membership, so a major challenge was to ensure that pre-randomisation procedures occurred quickly enough to allow the groups to begin in a timely manner.

In total we ran three separate groups. MBT-Ai group attendance was adequate, although 27% of participants randomised to active treatment attended no sessions at all. This perhaps reflected either an ambivalence about seeking help for self-harm even within a cohort already receiving treatment from CAMHS, or the impulsivity of this population. However, half of those randomised to the MBT treatment arm attended more than 50% of sessions, which is comparable to the completed RCT of MBT-A [13]. We were able to follow-up 50% of our participants at 12 weeks and retain most of these (42% total) at 24 weeks. Again, this is similar to reports from other recent trials of self-harm interventions for adolescents [43].

Both self-reported self-harm and self-harm related hospital use reduced over time. There was no difference between groups but as a pilot study it was not adequately powered to detect small effects. In addition, for this pilot, we placed no restriction on TAU. We did collect service information in order to characterise routine clinical care [29]. We subsequently noted that 26% of the TAU alone group received a 7-week Distress Tolerance group intervention, based on the principles of DBT. DBT for adolescents who self-harm showed the largest effect sizes in a recent meta-analysis [19] and there is considerable overlap between DBT and MBT. Furthermore, 19% of participants were referred from Tier IV services, whose staff had previously been trained in AMBIT [28]. TAU for some young people might therefore consist of mentalization-influenced intervention. Further consideration should be given to the control condition for any definitive trial.

However, further analysis using data from the whole sample gave promising signals that were consistent with mentalization theory. Emotion regulation, reflective function and the interaction between reflective function and pre- treatment self-harm emerged as significant predictors of a reduction in self-reported self-harm and reflective function alone predicted reduced hospital presentation for self-harm independent of randomisation. Furthermore, measures of social anxiety, emotion regulation and borderline features all also demonstrated change over time in the expected direction. Multi-level modelling further demonstrated that baseline self-harm, risk-taking and emotion regulation influenced change over time on both self-harm outcomes. Moreover, once these had been taken into account, self-reported mentalization had a bigger effect than the other variables. Additionally, multi-level modelling indicated that mentalization had an interaction effect with baseline self-harm. The emerging picture suggests that changes in mentalization may influence self-harm outcomes, warranting further investigation of the full MBT programme for this population as well as briefer mentalization-based interventions such as MBT-Ai in a fully powered definitive trial.

Another limitation is that we did not use an interview-based measure of self-harm. This was a decision informed largely by resource limitations, whilst acknowledging that different measurement methods for assessing self-harm result in widely disparate prevalence rates [18]. We did additionally assess self-harm related hospital use through examination of patient records. This ensured that we were able to collect at least one main variable for all participants who remained in the study.

We followed our adapted treatment manual which is available on request from the authors, ensuring replicability, and took steps to ensure fidelity to the manual and to principles of MBT. We report on attrition and, importantly, serious adverse events in addition to our main outcome variables relating to self-harm, providing a comprehensive account of any potential unwanted consequences of our intervention.

The other completed [13] and ongoing [44] trials of MBT-A offer far more intensive, multi-modal therapeutic programmes. A previous meta-analysis noted that those interventions with more substantial treatment ‘dosage’ were more successful [19]. On the other hand, the relative brevity of our intervention may have been a positive for many young people who may have been reluctant to participate in a year long programme. A second consideration is the degree to which an MBT intervention needs to deliver a skills based training versus a more traditionally therapeutic programme. One of our adaptations to the adult MBT manual was to include more experiential exercises and more focus on applying MBT skills in daily life, with psychoeducation remaining a significant component of our programme. It may well be that a more therapeutic focus, in group or individual format, is required, particularly for those with more entrenched difficulties. On the other hand, our skills based approach appeared well suited to our adolescent population, many of whom had complex presentations. The more efficient delivery minimises required service resources. In the UK, CAMHS are known to be under considerable pressure, stretched for resources and frequently struggling to meet demand. Comparatively brief interventions translate into feasible practice. Given that differential effect sizes seemed to indicate that interventions with strong family components worked best [19], a potential modification would be to incorporate MBT sessions for parents/caregivers. Clinically, it would seem important to ensure that the living environment of young people is primed to provide as best a ‘mentalizing culture’ as possible. How complexity and severity of presentation influences response to different formats and levels of intensity of MBT mode of delivery should be tested in future evaluations of MBT-A.

## Conclusions

Both study procedures and the intervention itself seemed feasible and acceptable, although a future trial would benefit from a distinct control condition. Self-reported self-harm and self-harm related hospital use reduced over the course of the intervention. In line with mentalization theory, reflective function was an independent predictor of reduced self-harm related hospital use at 12 weeks. Other variables also changed over time in the expected direction. Future research should seek to inform the required format and intensity of interventions offered.

## Data Availability

The datasets used and/or analysed during the current study are available from the corresponding author on reasonable request.

## Abbreviations

AMBIT: Adaptive Mentalization-Based Integrative Treatment
BPD: Borderline Personality Disorder
BPFSC: Borderline Personality Features Scale for Children
CAMH: Child and Adolescent Mental Health
CAMHS: Child and Adolescent Mental Health Services
CSRI: Client Service Receipt Inventory
DERS: Difficulties in Emotion Regulation Scale
ECRS-RC: Experiences in Close Relationships Scale–Revised Child version
ISM: Interpersonal Sensitivity Measure
MBT: Mentalization Based Therapy
MBT-A: Mentalization Based Treatment for Adolescents
MBT-Ai: Mentalization Based Treatment for Adolescents – Introductory group format
MBT-F: Mentalization Based Treatment – Family Therapy
MBTi: Mentalization Based Treatment – Introductory
NHS: National Health Service
RA: Research Assistant
RCADS: Revised Child Anxiety and Depression Scale
RCT: Randomised Controlled Trial
RFQ-Y: Reflective Functioning Questionnaire for Youths
RTSHI: Risk-Taking and Self-Harm Inventory
SPIRIT: Standard Protocol Items: Recommendations for Interventional Trials
TAU: Treatment as Usual
