# Correction to: Efficacy of Mentalization-based group therapy for adolescents: the results of a pilot randomised controlled trial

**DOI:** 10.1186/s12888-021-03102-8

**Published:** 2021-03-09

**Authors:** Helen Griffiths, Fiona Duffy, Louise Duffy, Sarah Brown, Harriet Hockaday, Emma Eliasson, Jessica Graham, Julie Smith, Alice Thomson, Matthias Schwannauer

**Affiliations:** 1 School of Health in Social Science, The University of Edinburgh, Old Medical School, Teviot Place, Edinburgh, EH8 9AG UK; 2 NHS Lothian Child and Adolescent Mental Health Services, Royal Edinburgh Hospital, Tipperlinn Road, Edinburgh, EH10 5HF UK

**Correction to: BMC Psychiatry (2019) 19:167**

**https://doi.org/10.1186/s12888-019-2158-8**

Following the publication of the original article [1], the authors identified an error in reference 27.

The incorrect reference:

27. Griffiths H, Duffy F, Duffy L, Brown S, Hockaday H, Eliasson E, Graham J, Thomson A, Happer R, Butler CM, et al. Efficacy of Mentalization-Based Group Therapy for adolescents: a pilot randomised controlled trail. Soc Sci Protoc. 2018;1:1–14. 10.7565/ssp20182647

The correct reference:

27. Griffiths H, Duffy F, Duffy L, Brown S, Hockaday H, Eliasson E, Graham J, Thomson A, Happer R, Butler CM, et al. Efficacy of Mentalization-Based Group Therapy for adolescents: a pilot randomised controlled trail. Soc Sci Protoc. 2018;1:1–14. 10.7565/ssp.2018.2647

The author group has been updated above and the original article [1] has been corrected.
